# Fine Mapping and Candidate Gene Identification of a White Flower Gene *BrWF3* in Chinese Cabbage (*Brassica rapa* L. ssp. *pekinensis*)

**DOI:** 10.3389/fpls.2021.646222

**Published:** 2021-05-07

**Authors:** Shuangjuan Yang, Xinxin Tian, Zhiyong Wang, Xiaochun Wei, Yanyan Zhao, Henan Su, Xiaobin Zhao, Baoming Tian, Yuxiang Yuan, Xiao-Wei Zhang

**Affiliations:** ^1^Institute of Horticulture, Henan Academy of Agricultural Sciences, Postgraduate T&R Base of Zhengzhou University, Zhengzhou, China; ^2^School of Agricultural Sciences, Zhengzhou University, Zhengzhou, China

**Keywords:** *Brassica rapa*, white flower, gene cloning, carotenoid, plastoglobule

## Abstract

Flower color is an important trait in plants. However, genes responsible for the white flower trait in Chinese cabbage are rarely reported. In this study, we constructed an F_2_ population derived from the Y640-288 (white flower) and Y641-87 (yellow flower) lines for the fine mapping of the white flower gene *BrWF3* in Chinese cabbage. Genetic analysis indicated that *BrWF3* was controlled by a single recessive gene. Using BSA-seq and KASP assays, *BrWF3* was fine-mapped to an interval of 105.6 kb. Functional annotation, expression profiling, and sequence variation analyses confirmed that the *AtPES2* homolog, *Bra032957*, was the most likely candidate gene for *BrWF3*. Carotenoid profiles and transmission electron microscopy analysis suggested that *BrWF3* might participate in the production of xanthophyll esters (particularly violaxanthin esters), which in turn disrupt chromoplast development and the formation of plastoglobules (PGs). A SNP deletion in the third exon of *BrWF3* caused the loss of protein function, and interfered with the normal assembly of PGs, which was associated with reduced expression levels of genes involved in carotenoid metabolism. Furthermore, we developed and validated the functional marker TXBH83 for *BrWF3.* Our results provide insight into the molecular mechanism underlying flower color pigmentation and reveal valuable information for marker-assisted selection (MAS) breeding in Chinese cabbage.

## Introduction

Flower color is one of the most important traits in plants, which provides a visual signal to attract insects for pollination (Kevan and Baker, 1983; Ariizumi et al., 2014). It also protects plants against disease and UV radiation and helps to maintain the normal physiological function of floral organs (Koes et al., 1994). In breeding, flower color can be used for identifying true/false hybrids and for evaluating seed purity in hybrid production (Zhang et al., 2018b). Carotenoids, flavonoids and betalains are three main classes of natural pigments contributing to different flower colors, among which the accumulation of carotenoids can cause yellow, orange and red colorations (DellaPenna and Pogson, 2006; Grotewold, 2006). In nature, greater than 800 structurally different carotenoid compounds have been identified (Nisar et al., 2015; Ding et al., 2019), which are further divided into two main groups, carotenes and xanthophylls (Nisar et al., 2015; Ding et al., 2019). In many cases, xanthophylls (i.e., letein, zeaxanthin, and violaxanthin) are the most prevalent carotenoids in yellow flowers (Ohmiya, 2011). The amount of carotenoids is a net result of biosynthesis, degradation and stable storage (Deruere et al., 1994; Li and Yuan, 2013; Ariizumi et al., 2014; Nisar et al., 2015; Yuan et al., 2015). Thus, factors that are associated with these processes act together to regulate the final carotenoid levels. Almost all of the genes and enzymes that catalyze the core reactions of carotenoid biosynthesis and degradation have been identified in plants (Yuan et al., 2015), whereas only a few genes have been reported to be involved in carotenoid sequestration and storage.

Carotenoids accumulate at high levels in chromoplasts, which possess a superior storage capacity to deposit carotenoids in carotenoid-lipoprotein sequestering structures, such as plastoglobules (PGs) (Li and Yuan, 2013; Yuan et al., 2015; Li et al., 2016). These structures contain carotenoids, polar lipids and carotenoid-associated proteins (van Wijk and Kessler, 2017). Carotenoids occupying the inner core interact with the acyl residues of polar lipids, which subsequently interact with carotenoid-associated proteins via polar head groups (Deruere et al., 1994; Vishnevetsky et al., 1999). Genes participating in the biogenesis of chromoplasts and the formation of carotenoid sequestration structures exert a strong effect on carotenoid metabolism in crops. For example, the *Or* gene, which encodes a DnaJ cysteine-rich domain-containing protein, triggers the differentiation of non-colored plastids into chromoplasts with an increased capacity to accumulate β-carotene in cauliflower and potato (Lu et al., 2006; Lopez et al., 2008). Either the *fibrillin* gene in pepper or the *CHRC* gene in cucumber, which encode carotenoid-associated proteins, is positively associated with carotenoid accumulation (Vishnevetsky et al., 1996; Pozueta-Romero et al., 1997). Overexpression of the pepper *fibrillin* gene in tomato increases the levels of carotenoids in fruits (Simkin et al., 2007). The *PYP1* gene in tomato, which is homologous to *PES2* (*PHYTYL ESTER SYNTHASE 2*) in *Arabidopsis*, plays a vital role in the production of xanthophyll esters in tomato anthers and petals (Ariizumi et al., 2014). Functional disruption of PYP1 converts flower color from yellow to pale yellow (Ariizumi et al., 2014). In pale-yellow-flowered petunia, the lower expression of *xanthophyll esterase* (*XES*) and lower proportions of esterified xanthophylls are the main reasons for low carotenoid accumulation (Kishimoto et al., 2019). Overexpression of *XES* from petals of *Ipomoea obscura*, tomato (*PYP1* gene), and marigold (*Tagetes erecta*) in this pale-yellow-flowered petunia all increases the esterified xanthophylls and causes deeper yellow coloration in flowers (Kishimoto et al., 2020).

In *Brassica* species, several genes controlling flower color have been reported. In *B.napus* and *B.oleracea*, the white flower trait is controlled by a single dominant gene, *carotenoid cleavage dioxygenase 4* (*CCD4*). A CACTA-like transposable element insertion in *CCD4* results in a petal color transition from white to yellow (Zhang B. et al., 2015; Han et al., 2019). In *B.juncea*, two recessive genes (*Bjpc1* and *Bjpc2*) control the white flower gene (Zhang et al., 2018a,b). These two genes, which are located on chromosomes A02 and B04, respectively, are homologous to *AtPES2* and participate in xanthophyll esterification (Zhang et al., 2018a,b). In *B.rapa*, the *carotenoid isomerase* (*BrCRTISO*) gene controls orange flower color as well as the orange coloration of the inner leaves of Chinese cabbage (Su et al., 2014; Zhang J. X. et al., 2015). Recently, Zhang N. et al. (2020) reported that the white flower trait in Chinese cabbage is controlled by two recessive loci, *Brwf1* and *Brwf2*. These two genes are located on chromosomes A01 and A09 and encode a plastid-lipid associated protein (PAP) and a carotenoid isomerase enzyme, respectively. Another study revealed that the white flower trait in *B.rapa* is controlled by a single recessive gene (Rahman, 2001). However, the gene underlying this white flower trait has not been reported thus far.

In this study, we conducted positional cloning of the white flower gene (*BrWF3*) in Chinese cabbage by using F_2_ populations derived from the white-flowered DH line ‘Y640-288’ and the yellow-flowered DH line ‘Y641-87’. The *BrWF3* gene was mapped to chromosome A02, and *Bra032957*, which is homologous to *AtPES2*, was identified as the candidate gene. Based on carotenoids profile analysis and transmission electron microscopy (TEM) analysis, as well as transcriptome analysis, the *BrWF3* gene was predicted to participate in carotenoids esterification and the biogenesis of PGs. A functional marker of *BrWF3* was also developed and validated in our study. This work will promote molecular marker-assisted selection (MAS) breeding and the exploration of molecular mechanisms that regulate flower color variation in Chinese cabbage.

## Materials and Methods

### Plant Materials

Two Chinese cabbage DH lines, white-flowered Y640-288 and yellow-flowered Y641-87, were used as parental lines to generate F_1_ and F_2_ populations for inheritance analysis and gene mapping. Additionally, three F_2_ populations, (Y640-288 × SD369)-F_2_, (Y640-288 × Chiifu)-F_2_, (Y66-83 × R16-11)-F_2_, were generated for marker validation by crossing the white-flowered DH lines Y640-288 and Y66-83 with the yellow-flowered DH lines SD369, Chiifu and R16-11. Furthermore, ten white-flowered and ten yellow-flowered DH lines (Supplementary Table 1) were also used to analyze mutations in the candidate gene. All the materials used in this study were provided by Institute of Horticulture, Henan Academy of Agricultural Sciences.

### Transmission Electron Microscopy (TEM) Analysis

Petals from Y640-288 and Y641-87 flowers at the flowering stage were used for transmission electron microscopy (TEM) analysis, which was performed according to Ariizumi et al. (2004).

### Carotenoid Identification and Quantification

Carotenoid composition was measured by MetWare (Wuhan, China). Petals from 10 white-flowered F_2_ plants were combined to form one replicate W-bulk and petals from 10 yellow-flowered F_2_ plants were included in the Y-bulk. In total, three replicates were assessed. Fresh petals were frozen in liquid nitrogen and stored at −80°C until needed. The direct extraction steps were performed according to Zhou et al. (2020). The saponified extraction steps were performed according to Inbaraj et al. (2008) with some modification. The direct and saponified extracts were then analyzed using an LC-APCI-MS/MS system (UHPLC, ExionLCs AD; MS, Applied Biosystems 6500 Triple Quadrupole). The chromatographic conditions and parameters for API 6500 Q TRAP LC-MS/MS System were performed as previously reported (Liu et al., 2020; Wang et al., 2020; Zhou et al., 2020). Specific procedures for extraction, identification and quantification of carotenoids were supported in Supplementary Material 1.

Carotenoids were identified by comparing their retention times and ion pair information (Supplementary Table 2). In saponified extracts, the integrated peak area was substituted into the linear equations of standard (Sigma, St. Louis, MO, United States) curves for content calculation (Supplementary Table 3); finally, the absolute content data for the carotenoids in the actual samples were obtained. Carotenoid content (μg/g) = B^∗^C/1000/D, where B is the concentration (μg/mL) obtained by substituting the integrated peak area of a carotenoid in the sample into the corresponding standard curve, C is the resuspension volume (μL), and D is the mass of the weighed sample (g) (Liu et al., 2020; Wang et al., 2020; Zhou et al., 2020).

### Bulked Segregant Analysis (BSA) by Resequencing

Using the modified cetyltrimethylammonium bromide (CTAB) method, total genomic DNA was isolated from young leaves of the parents and F_2_ (Y640-288 × Y641-87) plants (Liu et al., 2003). For BSA-seq, two DNA pools were constructed by mixing equal amounts of DNA from 50 white-flowered F_2_ individuals (W-pool) and 50 yellow-flowered F_2_ individuals (Y-pool). The Illumina HiSeq X Ten platform was used to generate 150-base paired-end reads for Y640-288, Y641-87, the W-pool and the Y-pool by Anoroad Biotech Co., Ltd. (Beijing, China). The raw data were deposited in the Sequence Read Archive (SRA) in NCBI as PRJNA682710.

The clean reads of Y640-288, Y641-87, the W-pool and the Y-pool were aligned to the *B.rapa* Chiifu reference genome version 1.5 using BWA software (Li and Durbin, 2010). SAMtools software package (version 1.3.1) (Li et al., 2009) was then used to call single-nucleotide polymorphism (SNP) and insertion/deletion (InDel) variants based on alignment files. To identify candidate regions associated with the white flower trait, the SNP-index and △(SNP-index) were calculated for all genomic positions in the W-pool and Y-pool. The SNP-index was estimated from the proportion of reads harboring SNPs among the entire number of reads compared to the reference genome sequence (Abe et al., 2012). Then △(SNP-index) was calculated by subtracting the SNP-index of the Y-pool from that of the W-pool (Takagi et al., 2013). We filtered out SNPs with SNP-index <0.3 or >0.8 simultaneously in the two bulked pools. Furthermore, SNPs with heterozygous genotypes in the parental lines were also excluded. A 1-Mb sliding window with a 50-kb increment was applied to slide across the genome, and Δ(SNP-index) graphs were plotted using the average Δ(SNP-index) against the positions of each sliding window. We calculated the statistical confidence intervals of Δ(SNP-index) among all SNP positions with given read depths under the null hypothesis of no major genes, and the 95% and 99% confidence intervals of the Δ(SNP-index) were then generated for each read depth according to Takagi et al. (2013).

### Kompetitive Allele-Specific PCR (KASP) Marker and Linkage Map Development

To map the *BrWF3* gene, we extracted the 70-bp upstream and downstream sequences of the selected SNP for KASP marker development. For each selected SNP, two allele-specific forward primers and one common reverse primer were designed using the Primer Premier 5.0 program (Singh et al., 1998) according to the standard KASP guidelines. The two allele-specific primers were added with the standard FAM (5′-GAAGGTGACCAAGTTCATGCT-3′) and HEX (5′-GAAGGTCGGAGTCAACGGATT-3′) tails at the 5′ end. The developed KASP markers were first validated in the two parental lines and F_1_ plants for polymorphism screening. Then, the polymorphic KASP markers (Supplementary Table 4) were employed to genotype the F_2_ population using 135 individuals. KASP assays were performed as described by Yang et al. (2020). The genetic linkage map was constructed using JoinMap 4.0^1^ software. Recombination values were converted into genetic map distances (cM) following the Kosambi mapping function (Kosambi, 1944).

### Cloning and Sequence Analysis of the Candidate Genes

To clone the DNA and cDNA sequences of the putative candidate genes, primers (Supplementary Table 5) were designed according to the *B.rapa* reference genome. PCR amplification was performed in a total volume of 25 μL according to the manual supplied with Phanta Max Master Mix (Vazyme Biotech Co., Ltd., Nanjing, China). The PCR products were sequenced by Sunya Biotech Co., Ltd. (Zhengzhou, China), and sequence alignments were performed using DNAMAN software. The complete coding sequences of candidate gene from Y641-87 and Y640-288 were submitted to GenBank under the accession numbers: MW362118 (Y641-87) and MW362119 (Y640-288), respectively.

### Quantitative Real-Time PCR (qRT-PCR)

Total RNA was extracted using the RNAprep Pure Plant Kit (Tiangen, Beijing, China) according to the manufacturer’s instructions. First-strand cDNAs were synthesized in a 20 μL volume containing approximately 7 μg RNA and oligo (dT) primers using the TransScript One-Step gDNA Removal and cDNA Synthesis Kit (Trans, Beijing, China). qRT-PCR was performed with 2 × TB Green Premix Ex Taq II (TaKaRa, Japan) on a Roche LightCycler 480-II System (Roche Applied Sciences, Beijing, China). The *Bractin* gene was used as an internal control (Fujimoto et al., 2006; Takada et al., 2019). Each qRT-PCR experiment was performed in triplicate and the resultant mean value was used for qRT-PCR analysis (Zhang et al., 2013). Relative expression levels were calculated using the 2^–Δ^ ^Δ^ ^Ct^ method (Livak and Schmittgen, 2001).

### Transcriptome Analysis

The W-bulk and Y-bulk each with three replicates used for carotenoid analysis were also used for transcriptome analysis. Six cDNA libraries were constructed and sequenced on the Illumina HiSeq X Ten platform at Metware Biotech Co., Ltd. (Wuhan, China). Raw reads were filtered by removing low-quality reads and reads containing the adapter or ploy-N using in-house Perl scripts available from Metware Biotech Co., Ltd. (Wuhan, China). The clean reads were aligned to the *B.rapa* V1.5 reference genome using HISAT2 software (Kim et al., 2015). Differentially expressed genes (DEGs) were identified using the DESeq2 package (v1.30.0) (Love et al., 2014). The *P*-value of the DEGs between samples was adjusted using the Benjamini & Hochberg method (Benjamini and Hochberg, 2000). Genes with an adjusted *P*-value ≤0.05 and | log2 (fold change)| ≥ 1 were recognized as DEGs. To determine the biological significance of the DEGs, Kyoto Encyclopedia of Genes and Genomes (KEGG) pathway enrichment analysis was implemented using KOBAS software (Wu et al., 2006).

## Results

### Phenotypic Characterization and Genetic Analysis of White Flowers in Chinese Cabbage

**FIGURE 1:**
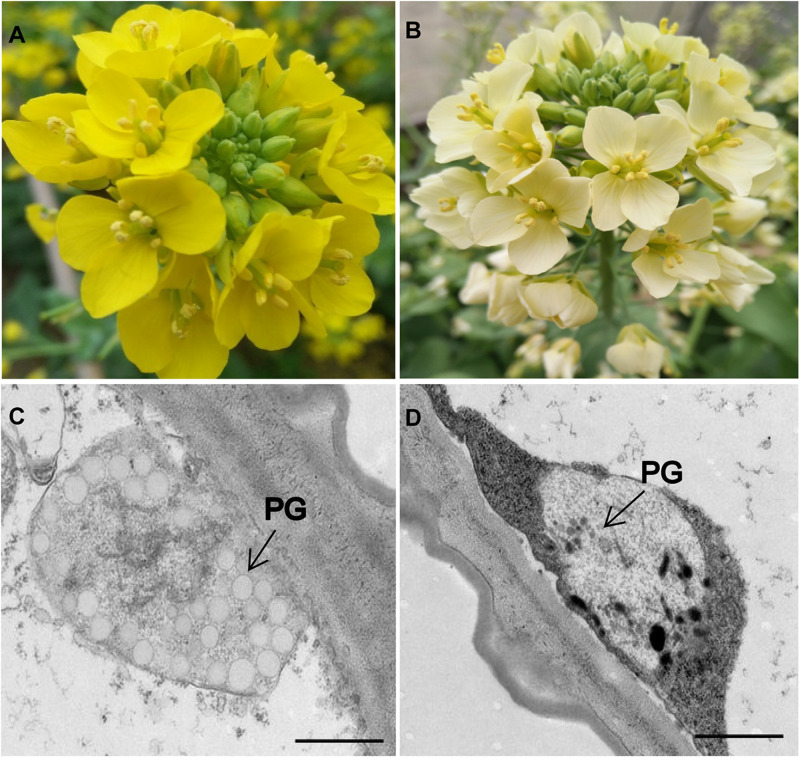
Flower coloration and chromoplast ultrastructure in the parental lines. **(A)** Yellow-flowered Y641-87. **(B)** White-flowered Y640-288. **(C,D)** Plastid ultrastructure in Y641-87 and Y640-288. PG, plastoglobule; Scale bar, 1 μm in **(C,D)**.

The phenotypic analyses showed significant differences in flower color between the two parental lines. In Y641-87, the flower organs, particularly the petal tissue, showed stable yellow coloration at the flowering stage, whereas those of Y640-288 showed white coloration (Figures 1A,B). The flower color in Y640-288 was traditionally called white color. It was not like white papers as in *B.napus* and *B.oleracea* (Zhang B. et al., 2015; Han et al., 2019), but similar to that in *B.juncea* (Zhang et al., 2018a,b), the flowers of which still had pale yellow pigments. TEM analysis showed that the petals of Y641-87 had normal chromoplasts with numerous fully developed PGs, whereas the petals of Y640-288 showed abnormal chromoplasts with only a few irregular and small PGs (Figures 1C,D).

To investigate the genetic inheritance of white flowers in Chinese cabbage, we performed reciprocal crosses between Y641-87 and Y640-288. The resulting F_1_ plants all displayed a yellow flower phenotype. The flowers of F_2_ plants exhibited two types of colorations, corresponding to the coloration of either Y641-87 or Y640-288. Among 200 F_2_ individuals, 142 exhibited yellow flowers, and 58 showed white flowers, corresponding to a segregation ratio of 3:1 by the Chi-square test (Table 1). In a larger F_2_ population, the segregation ratio was also 3:1 (1775 yellow:596 white, χ^2^ = 0.02). These results demonstrated that the inheritance of the white flower trait in Y640-288 follows a monogenic recessive pattern. We named this white flower gene *BrWF3*.

**TABLE 1:** Genetic analysis of the white flower trait in crosses between Y641-87 and Y640-288.

Population	Total	Yellow	White	Expected ratio	χ^2^	χ^2^*_0.05_*
P_1_(Y641-87)	10	10	0	–	–	–
P_2_(Y640-288)	10	0	10	–	–	–
F_1_ (Y640-288 × Y641-87)	15	15	–	–	–	–
F_1_ (Y641-87 × Y640-288)	15	15	–	–	–	–
F_2_	200	142	58	3:1	1.71	3.84

### Carotenoid Analysis in Yellow and White Flowers

**FIGURE 2:**
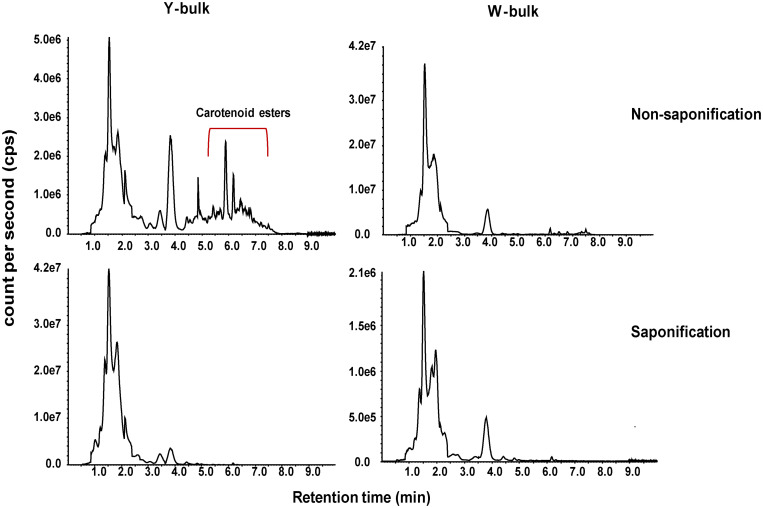
Total ion chromatograms of carotenoids in yellow and white petals. Carotenoids extracts from mature yellow **(left column)** and white **(right column)** petals were subjected to LC-APCI-MS/MS analysis under non-saponification (up row) and saponification (down row) treatments.

To investigate whether the lower pigmentation in white flowers was due to decreased carotenoid accumulation, we analyzed the carotenoid profiles of a white petal pool (W-bulk) and a yellow petal pool (Y-bulk). We detected 20 carotenoid peaks in the Y-bulk in the direct extracts (Figure 2 and Supplementary Table 6). Among these peaks, 9 peaks represented esterified carotenoids with retention times ranging from 5.5-7.5 min, as these peaks were not detected in the saponified sample (Figure 2). The esterified carotenoids are mostly derived from lutein and violaxanthin (Supplementary Table 6). In contrast, the composition and content of carotenoid esters in the W-bulk were much less than those in the Y-bulk (Figure 2). Analysis with saponification identified 10 carotenoids in both the W-bulk and Y-bulk (Figure 2 and Table 2). Two major xanthophylls, violaxanthin and lutein, accounted for approximately 83 and 91% of the total carotenoids in the Y-bulk and the W-bulk, respectively. The total average content of violaxanthin and all carotenoids in Y-bulk was about 2.76 and 1.70 times higher than that in the W-bulk, whereas lutein content did not significantly differ between Y-bulk and W-bulk (Table 2). Taken together, these results indicated that white petals accumulate less xanthophylls esters (likely violaxanthin esters) than yellow petals, resulting in lower carotenoid accumulation and color pigmentation.

**TABLE 2:** Carotenoid contents and compositions in Y-bulk and W-bulk after saponification.

Compounds	Concentration [μ g/g DW]	
	
	Y-bulk	W-bulk
**Carotenes**		
(E/Z)-Phytoene	118.58 ± 13.91^a^	21.71 ± 1.75**
β-Carotene	5.68 ± 0.12	5.71 ± 0.38^no^
α-Carotene	0.94 ± 0.10	0.23 ± 0.03**
**Xanthophylls**		
Violaxanthin	745.70 ± 35.64	269.76 ± 27.74**
Lutein	386.27 ± 11.94	464.52 ± 27.45^no^
Neoxanthin	69.34 ± 0.99	18.40 ± 1.24**
Zeaxanthin	22.4 ± 0.80	10.51 ± 0.50**
Antheraxanthin	9.96 ± 0.56	8.81 ± 0.79^no^
α-Cryptoxanthin	2.98 ± 0.24	1.58 ± 0.07**
β-Cryptoxanthin	2.65 ± 0.14	2.09 ± 0.17^no^
Total content of carotenes	125.2	27.64**
Total content of xanthophylls	1239.31	775.67**
Total content of carotenoids	1364.51	803.31**

^*a*^Mean ± SE (*n* = 3); ** Significant difference between Y-bulk and W-bulk within a component by Tukey’s test (*P* < 0.01); *Significant difference at *P* < 0.05; ^*no*^No significant difference at *P* < 0.05; n.d, not detected; DW: dry weight.

### The *BrWF3* Gene Is Located on Chromosome A02

To map the *BrWF3* gene, we conducted BSA-seq using two pooled samples, which comprised 50 white-flowered (W-pool) and 50 yellow-flowered (Y-pool) F_2_ plants, and two parental lines, Y640-288 and Y641-87. In total, 204, 209, 120, and 86 million raw data were generated for the W-pool, Y-pool, Y640-288 and Y641-87 (Supplementary Table 7), representing approximately 63-, 64-, 37- and 26-fold genome coverage, respectively, based on the estimated genome size of 485 Mb (Wang et al., 2011). The clean reads of each sample were mapped to the reference genome of the Chiifu cultivar. After filtering, a total of 358,141 SNPs and 54,500 InDels, which were distributed merely on ten chromosomes, were identified between the W-pool and the Y-pool. The Δ(SNP-index) of each position was calculated for sliding window analysis. According to the null hypothesis, a total of five adjacent regions on chromosome A02 (Supplementary Figure 1 and Supplementary Table 8) exhibiting significant linkage disequilibrium were identified as the candidate region for white flower trait at a 95% significant level. These results were not consistent with the assumption that the white flower trait is controlled by a single recessive nuclear genetic locus. However, most of the genomic regions on other chromosomes exhibited a Δ(SNP-index) = 0. In theory, the SNP-index of the W- and Y-pools should be the same in the genomic regions that are not related to the phenotypic diference (SNP-index = 0.5), and Δ(SNP-index) should equal to 0 (Islam et al., 2016; Wang et al., 2017). Therefore, the most likely chromosome where *BrWF3* located was A02.

### Fine Mapping of the *BrWF3* Gene

**FIGURE 3:**
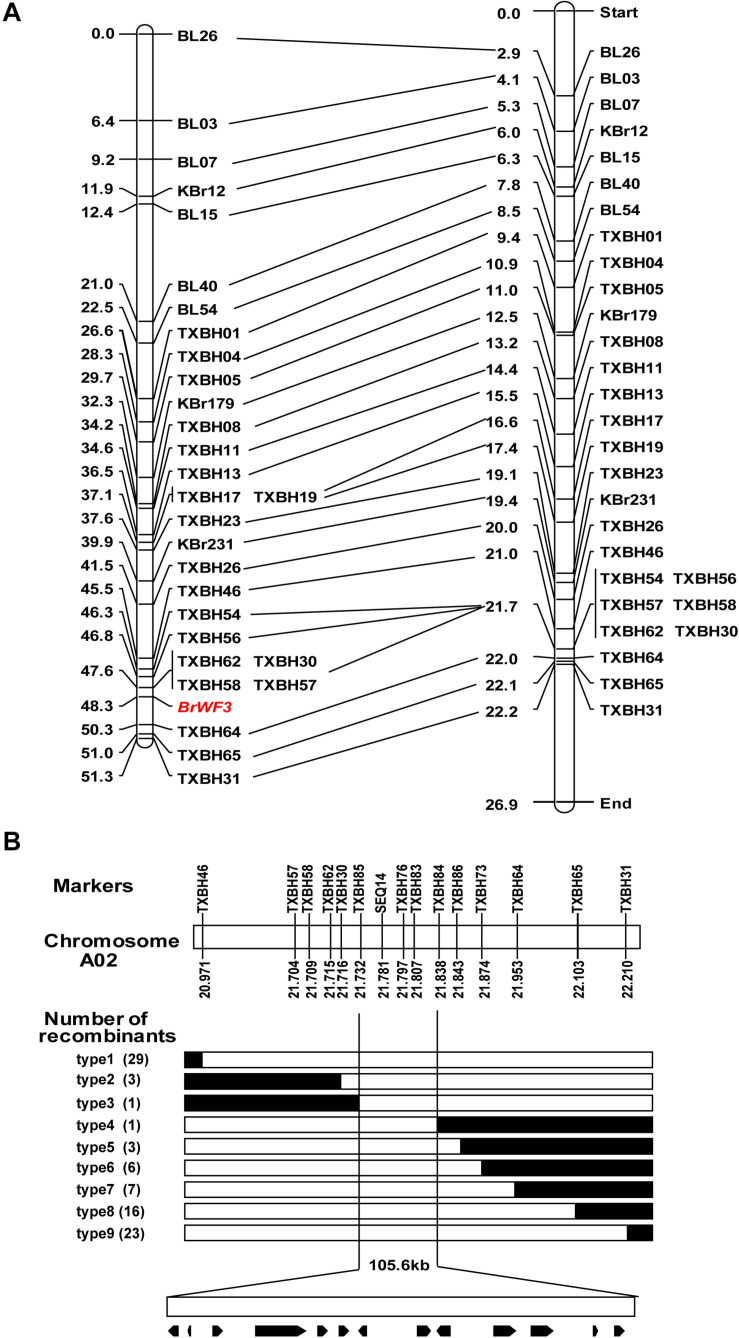
Initial and fine mapping of the *BrWF3* gene in Chinese cabbage. **(A)** Initial mapping of *BrWF3*. Genetic map of *BrWF3* is on the left, with cM as the unit. The corresponding physical map (right, unit: Mb) are also shown. **(B)** Fine mapping of *BrWF3*. The *BrWF3* gene was delimited to an interval between TXBH85 and TXBH84, with an estimated length of 105.6 kb, and 13 genes were annotated in this region based on the reference genome sequence. The genetic structure of each recombinant type is depicted as white for homozygous white flower, black for heterozygous alleles, respectively. The number of each recombinant type is indicated in the brackets.

Based on BSA-seq analysis, 46 KASP markers previously available in our group (Yang et al., 2020) and 65 newly developed KASP markers, which were uniformly distributed across chromosome A02, were used to identify polymorphisms between the two parental lines (Y640-288 and Y641-87). The results showed that 29 KASP markers (Supplementary Table 4) exhibited polymorphism. These polymorphic markers were further genotyped in 135 F_2_ plants for linkage analysis (Supplementary Table 9). The 135 F_2_ plants is a sub-set from the ‘whole’ population containing 200 plants in Table 1. The results revealed no recombinant individuals between *BrWF3* and markers TXBH57, TXBH58, TXBH62 and TXBH30 and 3 recombinant individuals between TXBH64 and *BrWF3*. The genetic distances between the *BrWF3* locus and TXBH30 and TXBH64 were 0.7 and 2.0 cM, respectively (Figure 3A). The order of the markers in the genetic map is consistent with that in the physical map (Figure 3A).

To fine-map the *BrWF3* locus, we screened 596 white-flowered F_2_ individuals using flanking markers (TXBH46 and TXBH31) and identified 52 recombinants. All the 52 recombinants were further genotyped using TXBH57, TXBH58, TXBH62, TXBH 30, TXBH64 and TXBH65, based on which 10 recombinants (type 2 and type 7) were identified (Figure 3B). Then, 21 new markers were developed, and seven of which exhibited polymorphism in the two parents (Supplementary Table 4). These seven new polymorphic markers were further used to screen all the 10 recombinants using KASP assay. The results delimited the *BrWF3* gene to a 105.6 kb interval between markers TXBH85 and TXBH84, each with one recombinant (type 3 and type 4) (Figure 3B). Three markers, SEQ14, TXBH76 and TXBH83, co-segregated with the *BrWF3* gene (Figure 3B).

### Candidate Gene Analysis

**FIGURE 4:**
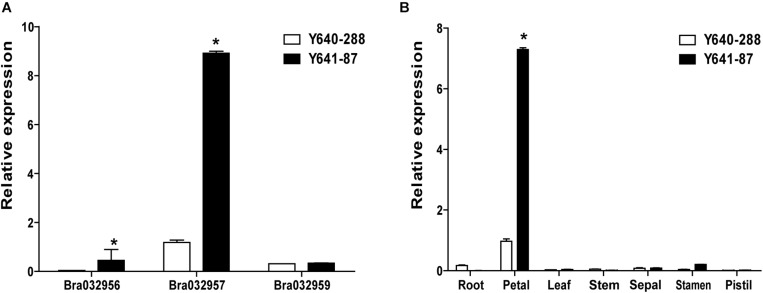
Gene expression data analyses. **(A)** Relative expression of *Bra032956*, *Bra032957*, and *Bra032959* in petals of the two parents. **(B)** Relative expression of *Bra032957* in different tissues of the two parents. Error bars represent the standard error of three biological replicates, and asterisks indicate significant differences (*t*-test, *p* < 0.05).

Based on the fine mapping results for *BrWF3*, DNA sequences in the 105.6 kb interval were analyzed in the *Brassica* database^2^ and comparative gene annotation in *Arabidopsis thaliana* was performed. As a result, 13 annotated or predicted genes were identified in the mapping region (Table 3). Four of these genes, *Bra032956*, *Bra032957*, *Bra032958*, and *Bra032959*, are homologs of *AT3G26840* (*PES2*) in *Arabidopsis thaliana*, which encodes a protein with phytyl ester synthesis and diacylglycerol acyltransferase activities and was previously reported to regulate carotenoid esterification (Zhang et al., 2018a,b; Kishimoto et al., 2020). Next, we examined the expression of these four candidate genes via RNA-seq and qRT-PCR analysis. RNA-seq analysis revealed that only *Bra032957* was differentially expressed among the four genes with the expression level decreasing approximately three fold in white petals compared with yellow petals (Supplementary Table 10). qRT-PCR analysis showed that the expression of *Bra032957* in petals was much higher than that of *Bra032956* and *Bra032959* and was significantly upregulated in yellow petals compared to white petals (Figure 4A). The results of qRT-PCR analysis and RNA-seq analysis were consistent. Subsequently, expression analysis in different tissues (root, stem, leaf, petal, sepal, stamen, and pistil) showed that *Bra032957* was predominantly expressed in petals (Figure 4B). Taken together, the results indicated that the *Bra032957* gene was the most likely candidate gene for *BrWF3*.

**TABLE 3:** Annotated genes in the candidate interval of the *BrWF3* locus.

Gene Name	Gene Position on A02	Arabidopsis thaliana homolog	Gene function
*Bra032947*	21730896-21732491	*AT3G26744*	MYC-like bHLH transcription factor
*Bra032948*	21734782-21735069	*AT3G26750*	HECT-like ubiquitin-conjugating enzyme (E2)-binding protein
*Bra032949*	21738466-21739774	*AT3G26760*	NAD(P)-binding Rossmann-fold superfamily protein
*Bra032950*	21743953-21753155	*AT3G17090*	Protein phosphatase 2C family protein;
*Bra032951*	21755514-21758199	*AT3G26770*	NAD(P)-binding Rossmann-fold superfamily protein
*Bra032952*	21762594-21764296	*AT3G26780*	Histidine phosphatase superfamily, clade-1
*Bra032953*	21768147-21770040	*AT3G26790*	B3 domain-containing transcription factor
*Bra032954*	21782268-21784649	*AT3G26810*	Auxin F box protein; AFB2
*Bra032955*	21786082-21787614	*AT5G14030*	translocon-associated protein beta(TRAPB) family protein
*Bra032956*	21797018-21801228	*AT3G26840*	Diacylglycerol acyltransferase; PES2
*Bra032957*	21805268-21810180	*AT3G26840*	Diacylglycerol acyltransferase; PES2
*Bra032958*	21833406-21833807	*AT3G26840*	Diacylglycerol acyltransferase; PES2
*Bra032959*	21836692-21839336	*AT3G26840*	Diacylglycerol acyltransferase; PES2

### Sequence Analysis of *Bra032957* as a Candidate Gene for *BrWF3*

**FIGURE 5:**
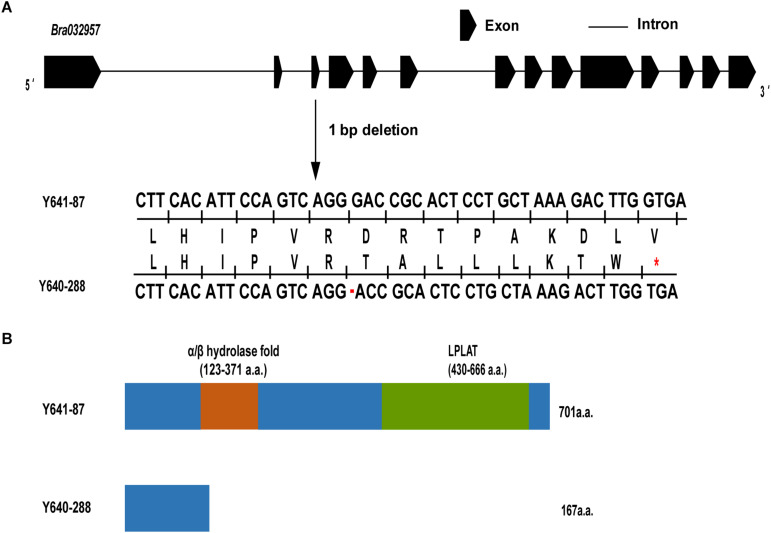
Gene structure, amino acid analysis and predicted protein structure of *BrWF3*. **(A)** *BrWF3* includes 14 exons and 13 introns. A single nucleotide deletion (G) in the third exon of white flower plants results in a premature stop codon (indicated with red asterisks) due to a frameshift mutation. **(B)** Protein structures of BrWF3 in the two parents. The SNP deletion in Y640-288 causes the loss of two conserved protein domains of BrWF3.

To characterize the sequence of the candidate genes in the white-flowered parental line Y640-288 and the yellow-flowered parental line Y641-87, the genomic sequence (gDNA) and coding sequence (CDS) of *Bra032957* were amplified and sequenced using specific primers (Supplementary Figure 2 and Supplementary Table 5). The results showed that the *Bra032957* gene of Y641-87 was 5162 bp in length and contained 14 exons and 13 introns. The CDS of the *Bra032957* gene in Y641-87 was 2106 bp in length. Sequence alignment revealed a base deletion (G) at 477 bp of the CDS in the third exon in Y640-288 (Supplementary Figure 3 and Figure 5A). The SNP deletion caused a frameshift mutation in the *Bra032957* gene and a premature stop codon in 168 a.a. residues (Figure 5A). Conserved domain analysis in NCBI showed that the *Bra032957* gene contained an α/β hydrolase-fold (amino acids 123-380) and a lysophospholipid acyltransferases (LPLAT) domain (amino acids 430-666), which can transfer acyl groups to acceptors, such as glycerol 3-phosphate (Figure 5B). The SNP deletion mutation caused a loss of the two conserved domains and ultimately caused the loss of function of the BrWF3 protein.

**FIGURE 6:**
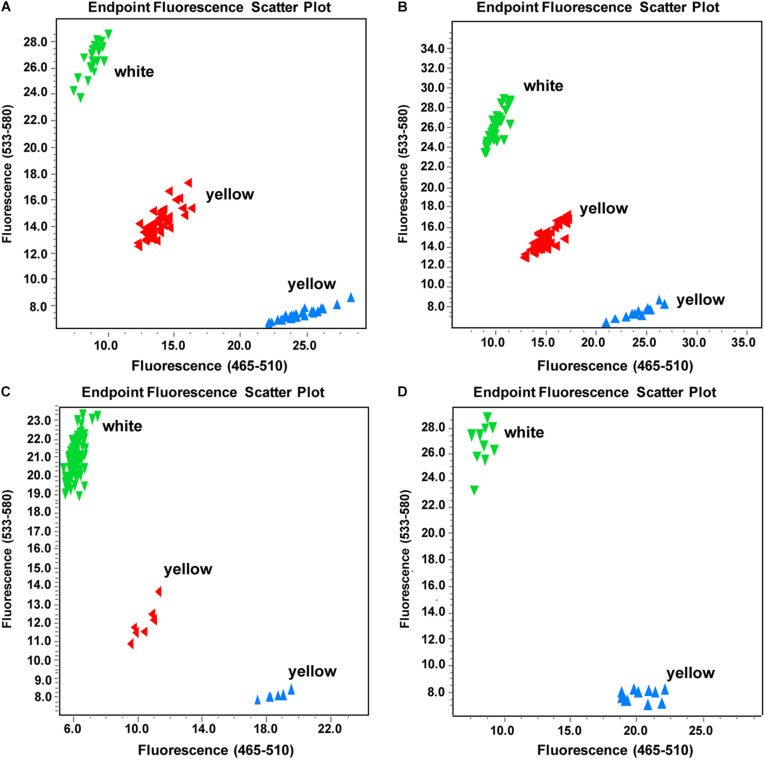
Genotyping results of marker TXBH83 in part individuals of different populations. **(A–D)** Genotyping results of TXBH83 in the (Y640-288 × Chiifu)-F_2_, (Y640-288 × SD369)-F_2_, and (Y66-83 × R16-11)-F_2_ populations and a natural population, respectively. More white-flowered plants were intentionally selected. The genotypes corresponding to those of Y640-288 are clustered to the Y axis, those matching Y641-87 genotypes are clustered to the X axis, and the heterozygous genotypes are clustered to the diagonal line. TXBH83 is totally co-segregated with the flower color phenotype.

Based on the identified SNP deletion, we designed a KASP marker TXBH83 to screen the other three F_2_ populations (Y640-288 × SD369-F_2_, Y640-288 × Chiifu-F_2_, Y66-83 × R16-11-F_2_), including a total of 282 individuals. The results showed that TXBH83 co-segregated with the flower color phenotype (Figures 6A-C and Supplementary Table 11). Furthermore, 10 white-flowered and 10 yellow-flowered DH lines were genotyped for TXBH83, which also showed a 100% consistency between the flower color phenotype and genotype (Figure 6D and Supplementary Table 11). Overall, these findings suggest that the *Bra032957* gene is the most promising candidate gene for the white flower gene *BrWF3* in Chinese cabbage.

### Transcriptome Analysis in Yellow and White Petals

**FIGURE 7:**
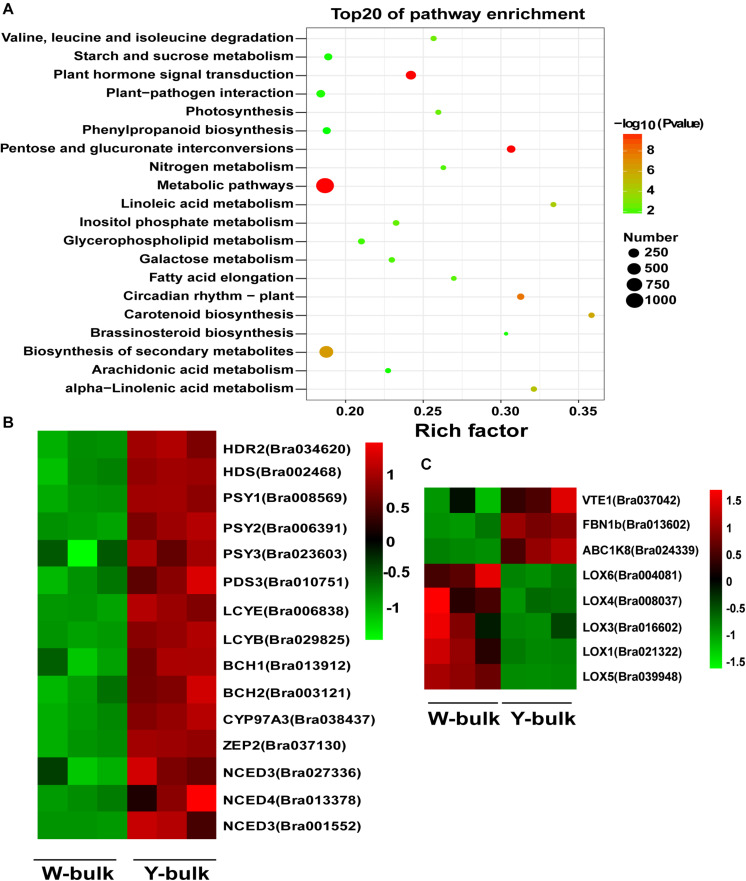
Transcriptome analysis in yellow and white petals. **(A)** Scatter plot of top 20 enriched KEGG pathways. Rich factor is the ratio of the DEG number to the background number in a certain pathway. The size of the dots represents the number of genes, and the color of the dots represents the range of the -log10(*p*-value). **(B)** Differentially expressed genes involved in carotenoid biosynthesis and degradation. **(C)** Differentially expressed genes associated with proteins in PGs. The heatmap colors are shown in log10(FPKM). Three biological replicates of the W-bulk and Y-bulk are shown.

To identify the gene regulatory networks involved in petal coloration, we performed comparative transcriptome analysis between W-bulk and Y-bulk. Approximately 300.8 million raw reads were generated for the six cDNA libraries, ranging from 43.1 to 57.4 Gb reads per library (Supplementary Table 12). All the raw reads were deposited in the NCBI Short Read Archive (SRA) database under accession number PRJNA682761. Among the clean reads, 85.2-87.46% were uniquely mapped to the reference genome (Wang et al., 2011). The Pearson correlation coefficients among the three replicates of each petal pool ranged from 0.98 to 0.99, indicating a high consistency among the three replicates (Supplementary Figure 4). In total, we identified 6,009 differentially expressed genes (DEGs) between the W-bulk and Y-bulk, among which 2,913 genes were up-regulated and 3,816 were down-regulated in the Y-bulk compared with the W-bulk. Pathway enrichment analysis based on the KEGG database revealed that carotenoid biosynthesis was the most significantly enriched pathway (Figure 7A). In the Y-bulk, genes involved in carotenoid biosynthesis (Figure 7B), such as *PSY (Bra006391* and *Bra008569*), *PDS* (*Bra010751*), *BCH1* (*Bra013912*) and *ZEP* (*Bra037130*), were significantly up-regulated. Genes involved in carotenoid degradation (Figure 7B), such as *NCED3* (*Bra027336* and *Bra001552*) and *NCED4* (*Bra013378*), were also upregulated. Pathways of linoleic acid metabolism, alpha-linolenic acid metabolism, glycerophospholipid metabolism and arachidonic acid metabolism were also enriched, and most of the genes in these pathways were downregulated (Figure 7A and Supplementary Table 12). For example, genes encoding glycerophosphodiester phosphodiesterase (*Bra040704*, *Bra027481*, *Bra035967*, *Bra020395*, *Bra002676*, and *Bra006785*), phospholipase A (*Bra015531* and *Bra010327*), phosphatidate phosphatase (*Bra029774*) were downregulated (Supplementary Figure 5 and Supplementary Table 13). However, genes participating in fatty acid elongation, such as 3-ketoacyl-CoA synthase (*Bra011936*, *Bra004513*, *Bra024749*, and *Bra004034*) and very-long-chain enoyl-CoA reductase (*Bra008657*), were significantly upregulated (Supplementary Figure 5 and Supplementary Table 13). These results suggested that saturated but not unsaturated fatty acids might be the main acyl group donors for esterification of xanthophylls. FIBRILLIN (FBN) and ACTIVITY OF BC1 COMPLEX KINASE (ABC1K) are the most abundant proteins in PGs (van Wijk and Kessler, 2017). In this study, *FBN1b* (*Bra013602*) and *ABC1K8* (*Bra024339*) were significantly upregulated in Y-bulk (Figure 7C). Furthermore, *FBN1b* is highly expressed. The average fragments per kilobase million (FPKM) value of *FBN1b* was as high as 8383 in Y-bulk, 3148 in the W-bulk. *VITAMIN E DEFICIENT 1* (*VTE1*), which encodes a key enzyme in tocopherol biosynthesis, was upregulated in Y-bulk but with no significant difference (Figure 7C and Supplementary Table 13). Lipoxygenase (LOX) are proteins recruited to chloroplast PGs and participated in jasmonate biosynthesis (van Wijk and Kessler, 2017). Genes encoding LOXs were all downregulated in yellow petals (Figure 7C and Supplementary Table 13), indicating that LOX proteins were not indispensible for PG development and formation in chromoplasts.

## Discussion

BSA-seq has been widely deployed for mapping agronomical traits in crops (Wang et al., 2017; Lee et al., 2020; Liang et al., 2020). In most cases, the genes controlling the target agronomic traits are located on the candidate regions detected by BSA-seq analysis. However, in one study, BSA-seq and traditional linkage analyses identified two different major loci for the purple leaf trait in *Brassica rapa*, one located on chromosome A07 and the other on A09 (Zhang X. et al., 2020). In our study, genetic analysis showed that the white flower trait in Y640-288 is controlled by a single recessive gene. However, our BSA-seq analysis identified five adjacent regions on A02, rather than only one sharp peak as noted in previous studies, and the results were not consistent with the genetic analysis. This phenomenon has also occurred in other studies from both our laboratory (unpublished data) and another laboratory (the Chinese cabbage research group at the Northwest A&F University). The two parental lines, Y640-288 and Y641-87, are both over-lapped head-type Chinese cabbage lines. While the reference material Chiifu-401-42 is a closed head-type Chinese cabbage line. Many significant structural variations were detected between the parental lines and the reference genome through single molecule real-time (SMRT) sequencing (Supplementary Figure 6). We suspected that the great structural variation between the parents and the reference genome might be responsible for the above discrepancy and reduce the efficiency of BSA-seq, which will be discussed in detail in a future study. Although the BSA-seq result was not perfect enough, the Δ(SNP-index) value for the other nine chromosomes were all close to 0, allowing the rapid mapping of the white flower gene on A02. The identified gene was named *BrWF3* because it was quite different from the other two genes reported previously (*Brwf1* on A01 and *Brwf2* on A09) (Zhang N. et al., 2020).

The present study successfully fine mapped the *BrWF3* gene to a physical interval of 105.6 kb. Functional annotation analysis of the 13 genes in the candidate region revealed that four genes, *Bra032956*, *Bra032957*, *Bra032958*, and *Bra032959*, which are homologous to *PES2* in *Arabidopsis*, might be candidate genes for *BrWF3*. In *Arabidopsis*, the *PES1* and *PES2* genes encode proteins that use medium-chain fatty acid-derived acyls to esterify phytyl released during chlorotic conditions. The resultant fatty acid phytyl esters accumulate in chloroplast PGs (Lippold et al., 2012). The *PYP1* (*Arabidopsis PES1* homolog) gene in tomato (Ariizumi et al., 2014) and the *Bjpc1* and *Bjpc2* (*Arabidopsis PES2* homolog) genes in *B.juncea* are all responsible for flower color changes (Zhang et al., 2018a,b). Furthermore, RNA-seq and qRT-PCR analysis revealed that only *Bra032957* was significantly and differentially expressed, showing considerably increased expression in yellow petals. We also examined the sequence variation between white and yellow petals. No sequence variation was found in the *Bra032958* gene. However, *Bra032956*, *Bra032957* and *Bra032959* each possessed one SNP variation. The SNPs in *Bra032956* (marker TXBH76) and *Bra032959* (marker TXBH84) occurred in intron regions, and only the SNP of *Bra032957* was located an exon. Additionally, we developed KASP markers for these three SNPs, namely, TXBH76 in *Bra032956*, TXBH83 in *Bra032957* and TXBH84 in *Bra032959*. The TXBH84 marker showed one recombinant according to fine mapping. Thus, the *Bra032959* gene could be excluded as a candidate. The TXBH76 and TXBH83 markers cosegregated with the phenotype during fine mapping. However, the TXBH76 marker could not be used to differentiate the yellow and white flower phenotypes in another F_2_ population (Y66-83 × R16-11) (Supplementary Figure 7). Accordingly, the possibility of *Bra032956* being the candidate gene was also eliminated. Moreover, we cloned the gDNA and cDNA sequences of *Bra032957* in Y641-87 and Y640-288. Sequence alignment revealed an SNP deletion in the third exon in the white flower parent Y640-288, which introduced a premature stop codon and caused enzymatic inactivity (Supplementary Figure 3). Taken together, the results indicated that the *Bra032957* gene was most likely responsible for the white flower trait in Chinese cabbage.

The KASP genotyping assay is one of the most efficient and cost-effective systems for SNP and small InDel genotyping (Semagn et al., 2013; Yang et al., 2020). In *B.juncea*, the white flower trait is collectively controlled by two recessive genes (*Bjpc1* and *Bjpc2*) (Zhang et al., 2018a,b). The *Bjpc1* gene, which is located on A02, is homologous to *Bra032956* in *B.rapa* (Zhang et al., 2018b). *Bjpc2* lies on B04 and is homologous to *Bra032957* in *B.rapa*. In our study, the *Bra032957* gene, which is located on A02, was the most promising candidate gene for the white flower trait in Chinese cabbage, suggesting a similar mechanism of flower color pigmentation in these two species. However, none of the closely linked and cosegregated markers of *Bjpc1* and *Bjpc2* showed polymorphisms in our F_2_ population (Y640-288 × Y641-87) (Supplementary Figures 8, 9), suggesting divergence between *B.juncea* and *B.rapa*. For the SNP deletion in *BrWF3*, we developed the KASP marker TXBH83, which completely cosegregated with the flower color phenotype in three other F_2_ populations and a small natural population. This marker can be used as a functional marker for MAS breeding and the assessment of genetic resources for developing new ornamental varieties with visual appeal, which has profound significance.

Using LC-APCI-MS/MS analysis with saponification, we observed that violaxanthin and lutein were the two main carotenoids in petals, accounting for approximately 83 and 91% of the total carotenoids in the Y-bulk and the W-bulk, respectively. These results were consistent with another study conducted in Chinese cabbage (Zhang N. et al., 2020) but differed from those of studies in tomato and *B.juncea* (Ariizumi et al., 2014; Zhang et al., 2018a,b), which suggested that the quantities and composition of xanthophylls in yellow flowers display considerable diversity among different species (Ohmiya, 2011). Notably, in the saponified extracts, the violaxanthin content in the Y-bulk was approximately 2.76 times higher than that in the W-bulk, and the total carotenoids content in the Y-bulk was about 1.70 times higher than that in the W-bulk, but the lutein content between these two bulks was not significantly different (Table 2). Thus, we hypothesized that the lower pigmentation in white flowers was due to the decreased carotenoid content, which is particularly related to the reduced violaxanthin content (but not lutein content). Furthermore, comparison of the carotenoid profile in direct and saponified extracts revealed increased proportion xanthophyll esters in yellow petals. Taken together, we proposed that the *BrWF3* gene played an important role in the production of xanthophyll esters (particularly violaxanthin esters) for yellow color pigmentation in Chinese cabbage and the lower pigmentation in white flowers was due to lower xanthophyll esters. Interestingly, among the direct extracts, in addition to the carotenoid esters detected in the Y-bulk, we also detected a few carotenoid esters in the W-bulk. While in tomato, carotenoid esters cannot be detected in pale yellow flowers (Ariizumi et al., 2014). The potential reasons for this difference might be associated with the whole-genome triplication of *B.rapa* (Wang et al., 2011). Although *BrWF3* encodes inactive proteins in white petals, other paralogs might express several active proteins participating in carotenoid esterification, which was confirmed by the expression data in our study (Figure 4).

In colored organs, such as petals and fruit, carotenoids accumulate mainly in chromoplasts, particularly in carotenoid-lipoprotein sequestering structures (i.e., PGs and fibrils) (Li and Yuan, 2013; Li et al., 2016; Yuan et al., 2015). These carotenoid-lipoprotein sequestering structures enhance the sink strength of chromoplasts (Yuan et al., 2015; Li et al., 2016). Deruere et al. (1994) proposed an architectural model for fibrils or PGs in which carotenoids were located in the inner core protected by polar lipids and carotenoid-associated proteins, the carotenoid core interacted with the acyl residues of the polar lipids, and the polar lipids then interacted with carotenoid-associated proteins via polar head groups. Carotenoids, polar lipids and carotenoid-associated proteins are three indispensable components of carotenoid-lipoprotein-sequestering structures. Many studies have revealed that genes encoding carotenoid-associated proteins are positively associated with carotenoid accumulation and that the mutations in these genes hamper PG formation (Vishnevetsky et al., 1996; Pozueta-Romero et al., 1997). In our study, TEM analysis showed that numerous fully developed PGs could be observed in yellow petals, whereas only a few irregular and small PGs could be observed in white petals, which was consistent with results obtained in *B.juncea* (Zhang et al., 2018a,b) and tomato (Ariizumi et al., 2014). We suspect that the *BrWF3* gene from our study, *PYP1* in tomato (Ariizumi et al., 2014), and *Bjpc2* in *B.juncea* (Zhang et al., 2018a), encoding proteins with phytyl ester biosynthesis and diacylglycerol acyltransferase activities, construct the interaction between carotenoids and the polar lipids by transferring the acyl group from polar lipids to the hydroxy (-OH) group of xanthophylls. An SNP deletion in *BrWF3* causes loss of protein function, thereby disturbing the connection between carotenoids and the polar lipids and further hampering the assembly and formation of PGs. As expected, comparative transcriptome analysis between the Y-bulk and W-bulk showed that the genes involved in three indispensable components of PGs, carotenoids, polar lipids and carotenoid-associated proteins, were downregulated in white petals, in which the development and formation of PGs were hampered.

## Conclusion

In the present study, we delimited the *BrWF3* gene responsible for the white flower trait in Chinese cabbage. BSA-seq and linkage analysis via KASP assays were employed to fine-map the *BrWF3* gene to an interval of 105.6 kb. Functional annotation analysis, expression analysis and sequence variation analysis revealed that *Bra032957*, which encodes a protein with phytyl ester synthesis and diacylglycerol acyltransferase activities, was the most likely candidate gene for *BrWF3*. *BrWF3* participated in the production of xanthophyll esters (particularly violaxanthin esters) and the formation of PGs. An SNP deletion in the third exon of *BrWF3* caused the loss of protein function and interfered with the formation of PGs, which subsequently reduced the activity of carotenoid metabolism and the content of carotenoids. Furthermore, we developed and validated the functional marker TXBH83 for *BrWF3*. These results not only provide valuable information for MAS breeding but also provide a significant contribution to research on the molecular mechanism underlying flower color pigmentation.

## Data Availability Statement

The datasets presented in this study can be found in online repositories. The names of the repository/repositories and accession number(s) can be found in the article/Supplementary Material.

## Author Contributions

SY conceptualized and designed the experiments and drafted the manuscript. XT and ZW performed the experiments and analyzed the data. BT, YY and X-WZ directed the whole study and provided the funding resource. XW, YZ, HS, and XZ participated in drafting the article and revising it critically. All authors contributed to the article and approved the submitted version.

## Conflict of Interest

The authors declare that the research was conducted in the absence of any commercial or financial relationships that could be construed as a potential conflict of interest.

## Footnotes

1 https://www.kyazma.nl/index.php/JoinMap/

2 http://brassicadb.org/brad/

## Abbreviations

BSA-seq: bulked segregant analysis coupled with whole-genome sequencing
CDS: coding sequence
DEGs: differentially expressed genes
DH: doubled haploid
FKPM: the fragments per kilobase of transcript per million
InDel: insertion-deletion
KASP: Kompetitive allele-specific PCR
KEGG: Kyoto encyclopedia of genes and genomes
MAS: molecular assisted selection
PGs: plastoglobules
qRT-PCR: Quantitative real time PCR
SNP: single nucleotide polymorphism
TEM: transmission electron microscopy
LC-MS/MS: liquid chromatography-tandem mass spectrometry.
