# Supplementary material for: Fine Mapping and Candidate Gene Identification of a White Flower Gene BrWF3 in Chinese Cabbage (Brassica rapa L. ssp. pekinensis)
Source: Front Plant Sci. 2021 May 7;12:646222. doi: 10.3389/fpls.2021.646222 (PMC8138439; doi:10.3389/fpls.2021.646222)
Supplement: Supplementary file 3 [file Data_Sheet_3.PDF]

Carotenoid Identification and Quantification

1. Sample preparation and extraction

Fresh petals were frozen in liquid nitrogen and kept at -80 ℃ until need. The direct extraction steps were performed according to Zhou et al. (2020): (1) A freeze-dried sample was removed from storage at ultralow temperatures and ground (30 Hz, 1 min) to a powder with a grinder (MM 400, Retsch, Haan, Germany). (2) A mixture of n-hexane: acetone: ethanol (1:1:2, v/v/v) was prepared as the extraction solution, and then 0.01% BHT (g/mL) and 50 mg of ground powder were mixed with an appropriate amount of the extraction solution (1 mL) and internal standard ([13C10] β-carotene, 20 μg/mL, 10 μL). (3) The mixture was vortexed at room temperature for 20 min. (4) The mixture was then centrifuged, and the supernatant was collected. (5) Steps (2), (3) and (4) were repeated, and the supernatants were combined and evaporated to dryness under nitrogen gas stream. (6) A mixture of methanol and methyl tert butyl ether (3:1, v/v) was prepared; the sample was resuspended with an appropriate amount the solution (100 μL), vortexed thoroughly until it was fully dissolved, and centrifuged. (7) After filtering through a 0.22 μm membrane, the sample was stored in an amber autosampler vial for LC-MS/MS analysis. The saponified extraction steps were performed according to Inbaraj et al. (2008) with some modification: after the first four steps as in direct extraction, saturated sodium chloride (NaCl) solution (1 mL) was added to the supernatants and vortexed, and the upper layer was collected, this step was repeated two times more. Then, the supernatant was evaporated to dryness and dissolved in MTBE (1 mL), then 10%KOH-MeOH (50 μL) was added, the mixture was vortexed, the reaction was allowed to take place at room temprature over night. After the reaction, saturated NaCl solution (1 mL) was added and vortexed, and the upper layer was collected, this step was repeated two times and the supernatant was evaporated to dryness and reconstituted in mixed solution of methanol: MTBE (100 μL). The solution was filtered through 0.22 μm filter for further LC-MS analysis.

2. Chromatographic conditions

Sample extracts were analysed using an LC-APCI-MS/MS system (UHPLC, ExionLC™ AD, https://sciex.com.cn/; MS, Applied Biosystems 6500 Triple Quadrupole, https://sciex.com.cn/). The analytical conditions were as follows: HPLC column, YMC C30 (3 μm, 2 mm × 100 mm); solvent system, methanol: acetonitrile (3:1, V/V) (0.01% BHT, 0.1% formic acid) and methyl tert-butyl ether (0.01% BHT); gradient programme, 100:0 V/V at 0 min, 100:0 V/V at 3 min, 58:42 V/V at 6 min, 20:80 V/V at 8 min, 5:95 V/V at 9 min, 100:0 V/V at 9.1 min, and 100:0 V/V at 11 min; flow rate, 0.8 mL/min; temperature, 28 ℃; injection volume, 2 μL (Zhou et al., 2020; Liu et al., 2020; Wang et al., 2020).

3. APCI-Q Trap-MS/MS

API 6500 Q TRAP LC-MS/MS System, equipped with an atmospheric pressure chemical ionization (APCI) Turbo Ion-Spray interface, operating in a positive ion mode and controlled by Analyst 1.6.3 software (AB Sciex), was used to detect metabolites eluted form the column. The APCI source operation parameters were as follows: ion source, APCI+; source temperature, 350 ℃; curtain gas (CUR) were set at 25.0 psi; the collision gas (CAD) was medium. Declustering potential (DP) and collision energy (CE) for individual MRM transitions were obtained with further DP and CE optimization. A specific set of MRM transitions were monitored for each period according to the carotenoids eluted within this period(Zhou et al., 2020; Liu et al., 2020; Wang et al., 2020).

Carotenoids were identified by comparing their retention times and ion pair information (**Supplementary Table S2**). In saponified extracts, the integrated peak area was substituted into the linear equations of standard (Sigma, St. Louis, MO, USA) curves for content calculation (**Supplementary Table S3**); finally, the absolute content data for the carotenoids in the actual samples was obtained. Carotenoid content (μg/g) = B*C/1000/D, where B is the concentration (μg/mL) obtained by substituting the integrated peak area of a carotenoid in the sample into the corresponding standard curve, C is the resuspension volume (μL), and D is the mass of the weighed sample (g) (Zhou et al., 2020; Liu et al., 2020; Wang et al., 2020) .

References

Inbaraj, B.S., Lu, H., Hung, C.F., Wu, W.B., Lin, C.L., and Chen, B.H. (2008). Determination of carotenoids and their esters in fruits of *Lycium barbarum* Linnaeus by HPLC-DAD-APCI-MS. *J. Pharm. Biomed. Anal.* 47, 812-818, doi: 10.1016/j.jpba.2008.04.001

Liu, Y., Lv, J., Liu, Z., Wang, J., Yang, B., Chen, W., et al. (2020). Integrative analysis of metabolome and transcriptome reveals the mechanism of color formation in pepper fruit (*Capsicum annuum* L.). *Food Chem*. 306: 125629. doi: 10.1016/j.foodchem.2019.125629

Wang, H., Huang, Y., Xiao, Q., Huang, X., Li, C., Gao, X., et al. (2020). Carotenoids modulate kernel texture in maize by influencing amyloplast envelope integrity. *Nature Communications* 11, 1-13. doi: 10.1038/s41467-020-19196-9

Zhou, W., Niu, Y., Ding, X., Zhao, S., Li, Y., Fan, G., et al. (2020). Analysis of carotenoid content and diversity in apricots (*Prunus armeniaca* L.) grown in China. *Food Chem*. 330:127223. doi: 10.1016/j.foodchem.2020.127223
